# RACIAL DISPARITIES IN THE ASSOCIATION BETWEEN VISION IMPAIRMENT AND FOOD INSECURITY IN THE US

**DOI:** 10.1093/geroni/igac059.947

**Published:** 2022-12-20

**Authors:** Varshini Varadaraj, Priyanka Kumar, Jessica Brinson, Jiangxia Wang, Laura Samuel, Adrienne Scott, Bonnielin Swenor

**Affiliations:** Johns Hopkins University, Baltimore, Maryland, United States; Johns Hopkins University School of Medicine, Baltimore, Maryland, United States; Howard University College of Medicine, Washington, District of Columbia, United States; Johns Hopkins Bloomberg School of Public Health, Baltimore, Maryland, United States; Johns Hopkins University, Baltimore, Maryland, United States; The Johns Hopkins Wilmer Eye Institute, Baltimore, Maryland, United States; Johns Hopkins University, Baltimore, Maryland, United States

## Abstract

We examined racial disparities in household food insecurity among low-income Americans with and without vision impairment in the National Health Interview Survey, years 2011-2018. Among 257,620 U.S. adults below a threshold of 150% poverty, 15% of White, 16% of Black, and 8% of Asian Americans, and 18% of American Indians and Inuits reported vision impairment. In analyses adjusted for sociodemographic variables, vision impairment was associated with >100% greater odds (95% CI=2.01-2.31) of 30-day food insecurity, as compared to no vision impairment. Further, odds of household food insecurity were higher among Black Americans (OR=1.37, 95% CI=1.29-1.47), and American Indians and Inuits (OR=1.38, 95% CI=1.15-1.66) than White Americans, while Asians had lower odds (OR=0.45, 95 %CI=0.36-0.57). These findings highlight that low-income adults with vision impairment and racial minorities experience food disparities and dietary inadequacy, an area of disadvantage that can influence overall health, in a nationally representative sample in the U.S.

